# Effect of Spatial Distribution of nZVI on the Corrosion of nZVI Composites and Its Subsequent Cr(VI) Removal from Water

**DOI:** 10.3390/nano12030494

**Published:** 2022-01-30

**Authors:** Yixuan Li, Shuangqiu Huang, Yaqin Song, Xinfang Zhang, Sijia Liu, Qiong Du

**Affiliations:** School of Engineering, China Pharmaceutical University, Nanjing 211198, China; liyixuan_1117@163.com (Y.L.); 2111904034@e.gzhu.edu.cn (S.H.); songyq_11@126.com (Y.S.); zhxf20@163.com (X.Z.); liusijia_98@163.com (S.L.)

**Keywords:** nanoscale zero-valent iron (nZVI), pre-corrosion, Cr(VI) adsorption and reduction, corrosion products

## Abstract

There have been many studies on contaminant removal by fresh and aged nanoscale zero-valent iron (nZVI), but the effect of spatial distribution of nZVI on the corrosion behavior of the composite materials and its subsequent Cr(VI) removal remains unclear. In this study, four types of D201-nZVI composites with different nZVI distributions (named D1, D2, D3, and D4) were fabricated and pre-corroded in varying coexisting solutions. Their effectiveness in the removal of Cr(VI) were systematically investigated. The results showed acidic or alkaline conditions, and all coexisting ions studied except for H_2_PO_4_^−^ and SiO_3_^2−^ enhanced the corrosion of nZVI. Additionally, the Cr(VI) removal efficiency was observed to decrease with increasing nZVI distribution uniformity. The corrosion products derived from nZVI, including magnetite, hematite, lepidocrcite, and goethite, were identified by XRD. The XPS results suggested that the Cr(VI) and Cr(III) species coexisted and the Cr(III) species gradually increased on the surface of the pre-corroded D201-nZVI with increasing iron distribution uniformity, proving Cr(VI) removal via a comprehensive process including adsorption/coprecipitation and reduction. The results will help to guide the selection for nZVI nanocomposites aged under different conditions for environmental decontamination.

## 1. Introduction

As an environmentally functional material, zero-valent iron (ZVI) has been widely used for in situ pollution remediation because of its high reactivity, low price, and environmental friendliness [1]. Compared with ZVI, nanoscale zero-valent iron (nZVI) has a higher specific surface area and high reactivity. Nevertheless, the weaknesses, including poor stability, difficult separation, and rapid passivation, remarkably limit the effectiveness of nZVI and its practical application [2]. To counteract these drawbacks, an effective approach is to immobilize nZVI into porous materials such as activated carbon [3], bentonite [4], kaolinite [5], chitosan [6], and exchange resin [7], which can be used as a carrier to support nZVI with good mechanical strength and adjustable pore structure.

ZVI and its modifications could effectively degrade or transform several water pollutants such as persistent pollutants [8], heavy metal [9,10,11], coloring dyestuff [12], and phosphate [13]. However, it is necessary to consider the corrosion of ZVI while focusing on the removal of contaminants, because there is a certain time interval between the synthesis and practical application of ZVI [1,14]. ZVI easily reacts with oxygen and water molecules that could consume electrons because of its high activity and low electron selectivity [15], leading to corrosion product iron oxides or hydroxides forming on the surface of ZVI, such as Fe_3_O_4_, Fe_2_O_3_, Fe(OH)_3_, and γ-FeOOH [16,17]. In other words, ZVI employed for practical environment remediation has been pre-corroded under different conditions. The presence of corrosion products might increase the non-reduction process. The degree to which the reactivity of ZVI decreases depends on the thickness and composition of the corrosion product on the ZVI surface [17,18,19]. Many factors affect the corrosion degree of ZVI, such as the solution chemistry, exposure time, and structure [20,21,22]. Nowadays, several studies have been carried out on the corrosion of ZVI nanoparticles in the process of pollutant removal [23,24]. Nevertheless, little is known about the influence of supported nZVI structure in different pre-corrosion conditions on the removal of contaminants. Therefore, systematic investigation is required to explore the effect of the pre-corrosion of supported nZVI with different structures and to provide a potential method to improve the decontamination performance of nZVI.

In this study, Cr(VI) was used as a probe to evaluate the performance of nanocomposite. A macroporous anion exchange resin (D201) was utilized as the support for nZVI to improve the dispersion, and the synthetic nanocomposite (D201-nZVI) was employed to remove Cr(VI) from water. We systematically investigated the removal performance of Cr(VI) by pre-corroded D201-nZVI with different iron distributions. Moreover, the influences on D201-nZVI under different pre-corrosion conditions (e.g., ions, initial pH, dissolved oxygen (DO), corrosion time, and humic acid) were studied on Cr(VI) removal. The chemical compositions of D201-nZVI before and after pre-corrosion were analyzed using X-ray diffraction (XRD) and X-ray photoelectron spectroscopy (XPS), respectively. In addition, the removal mechanism by the pre-corroded D201-nZVI was elucidated.

## 2. Materials and Methods

### 2.1. Materials and Chemicals

The D201 resin, styrene–diethylene benzene copolymer with quaternary ammonium group (mean diameter of 0.7–0.8 mm) was purchased from Hangzhou Zhengguang Technology, Co., Ltd. (Hangzhou, China). D201 was washed with 1.5 M NaOH, 1.5 M HCl, distilled water, and ethanol to remove the potential residual impurities and dried at 40 °C for 24 h before use. FeCl_3_·6H_2_O, MgCl_2_·6H_2_O, NaH_2_PO_4_·2H_2_O, Na_2_SiO_3_, and diphenylcarbazide were purchased from Sinopharm Chemical Reagent Co., Ltd. (Shanghai, China) K_2_Cr_2_O_7_, NaCl, CaCl_2_, Na_2_SO_4_, and NaOH were purchased from Xilong Science Co., Ltd. (Shantou, China). HCl, NaNO_3_, and NaHCO_3_ were purchased from Nanjing Chemical Reagent Co., Ltd. (Nanjing, China). KCl and KBH_4_ were purchased from Shanghai Lingfeng Chemical Reagent Co., Ltd. (Shanghai, China). Anhydrous ethanol was purchased from Shanghai Titan Scientific Co., Ltd. (Shanghai, China). All chemicals were of analytical grade and all solutions were prepared with ultrapure water.

### 2.2. Synthesis of D201-nZVI

Four types of D201-nZVI nanocomposites were synthesized using an ion exchange and liquid-phase reduction method, as reported in the previously published literature [25]. In brief, it can be summarized as follows: (1) 1 g D201 resin was added to 100 mL FeCl_3_–HCl solution (1 M FeCl_3_·6H_2_O, 1 M HCl, saturated NaCl and 10% ethanol), stirring for 6 h at 25 °C to achieve the preloading of Fe (III) onto the inner surface of D201 by ion exchange; (2) after washing with absolute ethanol, the resin preloaded-Fe (III) was reduced into nZVI with different distributions using 100 mL KBH_4_ solutions with mass concentrations of 0.9%, 1.8%, 3.6%, and 7.2%, named D1, D2, D3, and D4, respectively. The D201-nZVI nanocomposites were filtered with 50-mesh gauze and washed with deionized water. Black spherical D201-nZVI nanocomposites were dried in a vacuum oven (DZF-6020, Xinmiao, Shanghai, China) at 50 °C.

### 2.3. Pre-Corrosion of D201-nZVI

Here, 0.1 g D201-nZVI was added in the Erlenmeyer flask with 100 mL ultrapure water at 25 °C using a constant temperature shaker (Taicang Qiangle Experimental Equipment Co., Ltd., Suzhou, China). The corrosion time was set 0, 24, 48, and 120 h, respectively. Moreover, pre-corroded D201-nZVI under different pre-corrosion conditions (e.g., ions, initial pH, dissolved oxygen (DO), corrosion time, and humic acid) were synthesized. The initial pH of the corrosion solution was adjusted to 3, 6, and 9 with 1 M HCl or 1 M NaOH. The oxygenated corrosion solution with DO content of 8.4 mg/L and deoxidizing solution with DO content of 1.6 mg/L were prepared as corrosion solution. The coexisting ions of the corrosion solution include 10 mmol/L NaCl, 10 mmol/L Na_2_SO_4_, 10 mmol/L NaNO_3_, 10 mmol/L NaHCO_3_, 10 mmol/L KH_2_PO_4_, and 10 mmol/L Na_2_SiO_3_, and the mixture of above anions or 10 mmol/L NaCl, 10 mmol/L KCl, 10 mmol/L CaCl_2_, and 10 mmol/L MgCl_2_, and the mixture of above cations), respectively (considering the Cr(VI) removal had insignificant effects with the increase of ion strength to 100 mmol/L (Figure S1), we set the ionic strength as 10 mmol/L in coexisting ions tests). The humic acid concentrations of corrosion solution were set to 0, 20, 40, and 100 mg/L, respectively. After pre-corrosion, the pre-corroded D201-nZVI were filtered and transferred to a clean Erlenmeyer flask for the batch experiments.

### 2.4. Batch Experiment

In the static experiment, a total of 0.1 g pre-corroded D201-nZVI under different solutions was added to 100 mL of 150 mg/L Cr(VI) solution and shaken at 25 °C for 24 h. In the kinetics experiment, 0.1 g pre-corroded D201-nZVI from pure H_2_O (corrosion time of 0 and 48 h) was added to a three-necked flask containing 500 mL of 30 mg/L Cr(VI) and stirred at 160 rpm. The Cr(VI) solution was sampled and analyzed several times. All experiments were performed in triplicate.

### 2.5. Material Characterization

Images of the iron distribution of fresh D1, D2, D3, and D4 were obtained using scanning electron microscopy (SEM, Quanta 400 FEG, FEG, OR, USA) with energy diffraction spectrum (EDS, Bruker, Karlsruhe, Germany) analyses. The BET surface areas of the fresh D1–D4 were measured using the N_2_ adsorption and desorption test (ASAP 2460, Micromeritics, Norcross, GA, USA). XPS (Thermo Scientific ESCALAB 250Xi, Thermo Scientific, Boston, MA USA) with a monochromatized Al Kα X-ray source was used to analyze the pre-corroded D201-nZVI in the H_2_O system after the Cr(VI) removal and the results were processed using XPSPEAK 41 software (version: 4.1). The X-ray diffraction (XRD) patterns of fresh D201-nZVI and pre-corroded D201-nZVI in the different coexisting solutions were recorded using a D8 ADVANCE XRD system (D8 Bruker Adv., Oberkochen, Germany).

### 2.6. Analytic Methods

Fe(III) was measured with phenanthroline colorimetry using a 752 UV−Visible spectrophotometer (YOKE Instrument, Shanghai, China) at 510 nm. Then, 0.1 g D201-nZVI was added to 50 mL HNO_3_ solution with a 10% mass concentration and reacted at 25 °C for 12 h [25]. The four types of D201-nZVI have almost the same iron content. The iron contents of D1, D2, D3, and D4 were determined to be 13.81%, 13.36%, 14.05%, and 13.75%, respectively. The concentration of Cr(VI) was measured with diphenylcarbazide colorimetry using a 752 UV−Visible spectrophotometer at 540 nm [26].

## 3. Results and Discussion

### 3.1. Characterization of D201-nZVI

**Figure 1:**
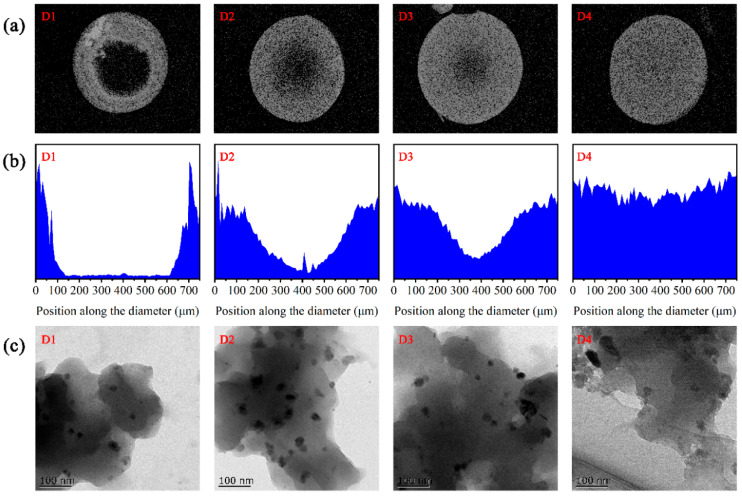
SEM-EDS spectra of elemental Fe mapping (**a**) and lines (**b**) in the cross section, and TEM images (**c**) of fresh D1, D2, D3, and D4.

As demonstrated in Figure 1a,b, the image of D1 showed the circular distribution of iron in the periphery of the spherical resin particles, as visualized through the SEM-EDX of iron element along the cross section and diameter of fresh D1–D4 samples. With the increase in reductant (KBH_4_) concentration from 0.9% to 7.2%, the iron distribution of D201-nZVI gradually shifted from the outside of the resin to the core area, indicating that the uniformity of the iron distribution in the resin notably improved from D1 to D4. Figure 1c shows the TEM image of fresh D1–D4 samples, which permitted the direct observation of microstructural features of this material. It could also be seen the details of four types of D201-nZVI at 100 nm that demonstrated nanoscale iron were well dispersed on the surface and interior of the D201 resin. The specific surface area was 9.75, 14.70, 15.63, and 16.40 m^2^ g^−1^ for D1, D2, D3, and D4, respectively (Table S1). For the D1–D4 composites, the increase in Fe distributions’ uniformity resulted in a significant increase in BET surface area, possibly due to the loading of the nZVI with higher specific surface areas. A more uniform distribution of nZVI inside the nanocomposite was favorable to restrict the aggregation of freshly formed nZVI. They could provide a more accessible surface and, thereby, increase the BET surface area.

**Figure 2:**
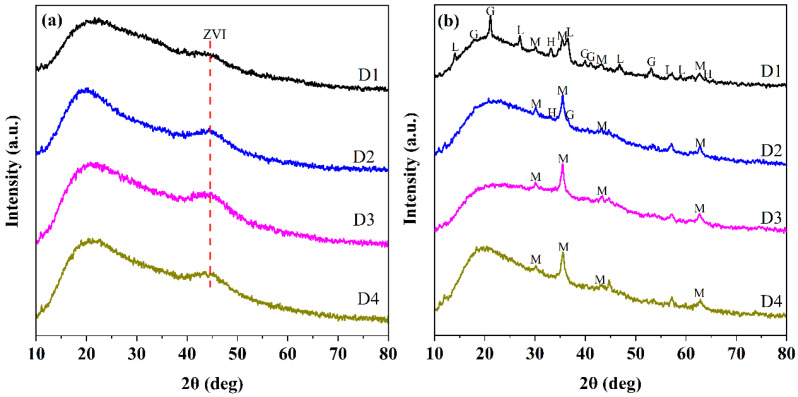
XRD patterns of fresh D201-ZVI (**a**) and D201-nZVI pre-corroded in the Cl^−^ system (**b**) (M: magnetite (Fe_3_O_4_); H: hematite (Fe_2_O_3_); L: lepidocrocite (γ-FeOOH); G: goethite (α-FeOOH).

The chemical components of D201-nZVI were further identified by XRD analysis. The XRD patterns for fresh D1, D2, D3, and D4 are shown in Figure 2a. The broad peaks at 44.8° were observed in the all types of D201-nZVI XRD patterns, indicating a small particle size and the existence of crystalline metallic Fe^0^ (JCPDS 06-0696) [1,7]. There was no significant difference in the peak of ZVI in the four types of fresh D201-nZVI, which might be caused by the dispersion of ZVI and poor crystallinity of D201-nZVI owing to the existence of a resin skeleton. However, the peak of Fe^0^ decreased with the corrosion process, indicating the oxidation and dissolution of ZVI. Different corrosion products in the four types of pre-corroded D201-nZVI were confirmed by XRD. For pre-corroded D3 and D4, the identified corrosion product was magnetite (Fe_3_O_4_, JCPDS19-0629). Besides magnetite, hematite (Fe_2_O_3_, JCPDS33-0664) was also identified in the pre-corroded D2 system. For pre-corroded D1, the dominant components were proved to be lepidocrocite (γ-FeOOH, JCPDS44-1415) and goethite (α-FeOOH, JCPDS29-0713), which were most heavily oxidized in the Cl^−^ system (Figure 2b) [1]. This implied that the particles had undergone severe oxidation during the pre-corrosion process.

### 3.2. Cr(VI) Removal Kinetics of the D201-nZVI before and after Corrosion

The removal properties of fresh and pre-corroded D201-nZVI toward Cr(VI) were systematically studied. Considering that the removal process involving nZVI contains an adsorption step resulting in an equilibrium effect, two kinetic models, pseudo-first-order and pseudo-second-order, were adopted to analyze the kinetics of the fresh and pre-corroded D201-nZVI. The non-linear forms of pseudo-first-order or pseudo-second-order kinetic equations could be followed as shown in Equations (1) and (2), respectively [27,28].

(1) $$Q_{t}=Q_{e}{(1\;-\;e}^{{-k}_{1}t})$$

(2) $$Q_{t}=\frac{k_{2}Q_{e}^{2}t}{{1+k}_{2}Q_{e}t}$$

where Q_e_ and Q_t_ are the adsorption capacity of Cr(VI) (mg/g) at the equilibrium and time t, respectively, and k_1_ and k_2_ are the pseudo-first-order and pseudo-second-order rate constants, respectively.

**Figure 3:**
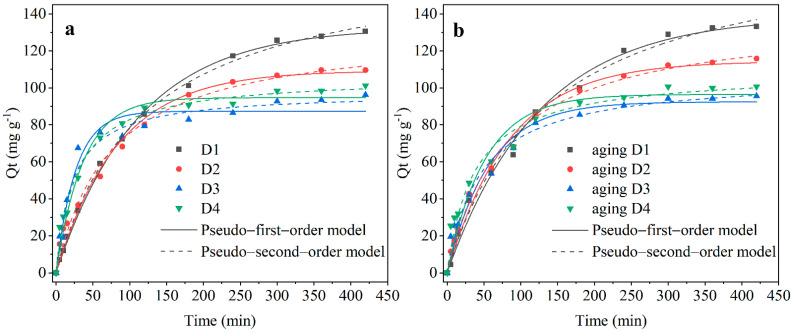
The kinetics of the pseudo-first-order and pseudo-second-order model for Cr(VI) removal by fresh D201-nZVI (**a**) and pre-corroded D201-nZVI after aging for 48 h (**b**).

It can be seen from Figure 3 that the removal rates of Cr(VI) of all four types of fresh and pre-corroded D201-nZVI increased with the corrosion time within the first three hours, following the order of D4 > D3 > D2 > D1. However, after reaching the equilibrium, the removal efficiency of Cr(VI) followed the order of D1 > D2 > D4 > D3. Although the removal rate of D1 was slow, the capacity at equilibrium was relatively high. D1 exhibited the best removal property before and after corrosion. In general, the adsorption capacity of pre-corroded D201-nZVI with same iron distribution was slightly larger than that of fresh D201-nZVI (Table 1). Figure 3 also showed the non-linear fitting plots of pseudo-first-order and pseudo-second-order kinetic models for Cr(VI) removal by fresh and pre-corroded D201-nZVI. The fitting parameters (Table 1) showed that two models could both generally fit the removal kinetic data (R^2^ > 0.94). The correlation coefficient R^2^ of the pseudo-second-order kinetic model was higher than that of the pseudo-first-order kinetic model. However, the fitted adsorption capacity of Cr(VI) at the equilibrium derived from the pseudo-first-order model was closer to the experimental values. Liu [29] also reported similar results. This indicated that the Cr(VI) removal process was consistent with the pseudo-first-order kinetic model for this system. The rate constants of pseudo-first-order (k_1_) with D1 to D4 after aging 48 h were 8.01 × 10^−3^, 1.18 × 10^−2^, 1.75 × 10^−2^, and 1.95 × 10^−2^ min^−1^, respectively. After corrosion, the adsorption capacities were 134.61, 116.51, 97.70, and 104.21 mg/g for D1, D2, D3, and D4, respectively, indicating that the generation of corrosion products might be beneficial to increase the adsorption properties of pre-corroded D201-nZVI.

### 3.3. Effects of Different Corrosion Conditions on the Cr(VI) Removal

#### 3.3.1. Corrosion Time

**Figure 4:**
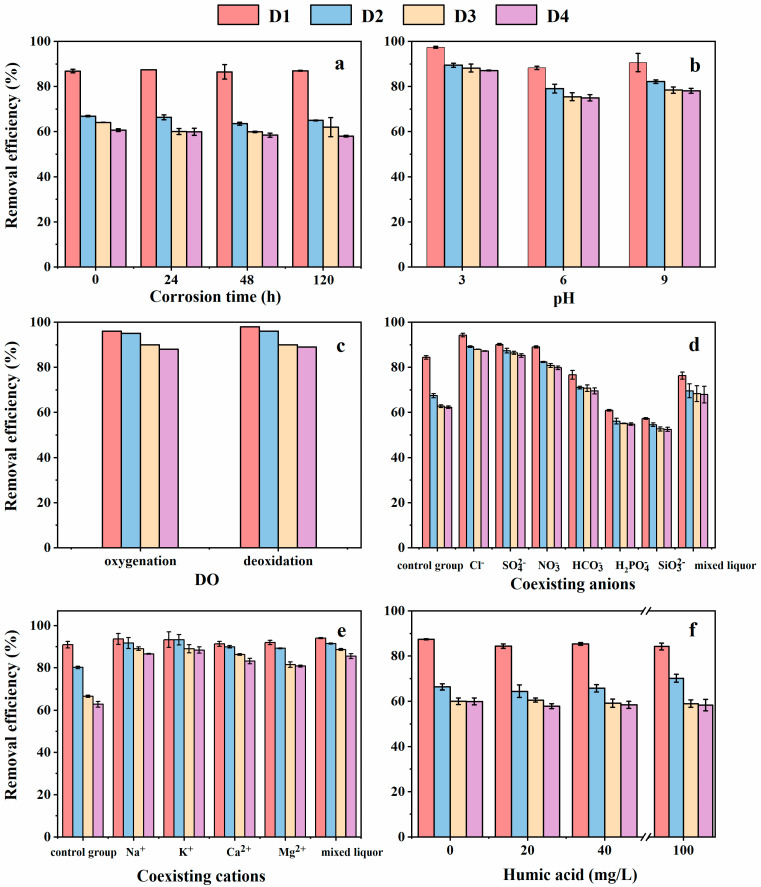
Effect of the corrosion time (**a**), initial pH (**b**), dissolved oxygen (**c**), coexisting anions (**d**), coexisting cations (**e**), and humic acid (**f**) on Cr(VI) removal by pre-corroded D201-nZVI.

Corrosion time had no significant effect on the Cr(VI) removal (Figure 4a). In the corrosion process, the reduction of ZVI was weakened owing to the oxidation of ZVI in the water. Meanwhile, the adsorption of iron oxide/hydroxide was enhanced with the formation of corrosion products. The reduction of Cr(VI) with ZVI and coprecipitation of Fe(III) and Cr(III) could be expressed as shown in Equations (3) and (4) [30]. The effects of reduction and absorption were partly offset, so there was no obvious difference in Cr(VI) removal efficiency at the different corrosion times. The Cr(VI) removal efficiency by the four types of D201-nZVI followed the order of D1 > D2 > D3 > D4 at the same corrosion time (Figure 4a). This differed from the result obtained for the removal kinetics, which might have resulted from the insignificant differences between pre-corroded D3 and D4 on Cr(VI) removal.

(3) $${\mathrm{CrO}}_{4}^{\;2-}+{\mathrm{Fe}}^{0}+8^{H+}\to {\mathrm{Fe}}^{3+}+{\mathrm{Cr}}^{3+}+4H_{2}O$$

(4) $${(1-x)\mathrm{Fe}}^{3+}+(x){\mathrm{Cr}}^{3+}+2H_{2}O\to {\mathrm{Fe}}_{\left(1-x\right)}{\mathrm{Cr}}_{x}\mathrm{OOH}↓+\;3H^{+}$$

Four types of D201-nZVI had almost the same iron content. However, nZVI in the D1 sample was primarily distributed on the outside of the resin, meaning that more iron oxide might be produced. The intensity of the iron oxide peaks in the pre-corroded D1 was stronger than that of pre-corroded D2, D3, and D4, confirming that the adsorption of iron oxide in the pre-corroded D1 system was better. This might explain why the removal efficiency followed the order of D1 > D2 > D3 > D4 (Figure 4a).

#### 3.3.2. Initial pH

As shown in Figure 4b, the removal efficiency increased in the order of pH 3 > pH 9 > pH 6. Previous studies have demonstrated that acid could dissolve the oxides on the surface of ZVI nanoparticles under acidic conditions, slowing down the corrosion of ZVI and maintaining a fresh Fe^0^ surface [31], which might be beneficial to improve the reduction of pre-corroded D201-nZVI to promote the Cr(VI) removal. This might explain why pre-corroded D201-nZVI exhibited the best removal efficiency under acidic conditions. Under alkaline conditions, ZVI could transform into oxide or hydroxide to promote the production of γ-FeOOH. Pre-corroded ZVI generally had a stratified structure including three layers: inner core of Fe^0^, outer shell of γ-FeOOH, and middle layer of Fe_3_O_4_/FeO. The Fe_3_O_4_ in the middle layer tend to gradually oxidize to Fe(OH)_3_ or γ-FeOOH in the presence of O_2_, as shown in Equations (5) and (6) [32]. γ-FeOOH showed a strong absorption of Cr(VI), which might be the reason that Cr(VI) removal by pre-corroded D201-nZVI was better in alkaline solutions. The Cr(VI) removal followed the order of D1 > D2 > D3 > D4 at the same initial pH, which was consistent with the above-mentioned conclusion (Figure 4b).

(5) $${4\;\mathrm{Fe}}_{3}O_{4}+O_{2}+18H_{2}O\;\to {\;12\;\mathrm{Fe}(\mathrm{OH})}_{3}$$

(6) $${4\;\mathrm{Fe}}_{3}O_{4}+O_{2}+6\;H_{2}O\;\to \;12\gamma -\;\mathrm{FeOOH}$$

#### 3.3.3. Dissolved Oxygen

There was insignificant difference in Cr(VI) removal by pre-corroded D201-nZVI in aerobic and anaerobic systems (Figure 4c), which confirmed that DO had little effect on the Cr(VI) removal. The Cr(VI) removal involved adsorption and reduction. High DO levels prompted the formation of iron oxide or hydroxide on the surface of pre-corroded D201-nZVI, which weakened the electron transport from Fe^0^ to Cr(VI). Oppositely, low DO levels prompted the reduction of pre-corroded D201-nZVI, which weakened the formation of iron oxide or hydroxide. The corrosion of ZVI has been well documented and the corrosion process is shown in Equations (7) and (9) [33]. These effects were partly offset, so there was no obvious difference in Cr(VI) removal efficiency in aerobic and anaerobic systems. The removal efficiency of Cr(VI) by pre-corroded D201-nZVI followed the order of D1 > D2 > D3 > D4 at the same DO concentration (Figure 4c).

2Fe^0^ + 2H_2_O + O_2_ → 2Fe^2+^ + 4OH^−^ (7)

4Fe (OH)_2_ + O_2_ + 2H_2_O → 4Fe(OH)_3_ (8)

4Fe^2+^ + O_2_ + 4H^+^→ 4Fe^3+^ + 2H_2_O (9)

#### 3.3.4. Coexisting Ions

Previous studies demonstrated that coexisting ions were adsorbed on the ZVI surface and had an effect on the corrosion evolution through multiple mechanisms [32]. In this study, we explored the effects of coexisting ions on the Cr(VI) removal by pre-corroded D201-nZVI. Generally, the effect of coexisting anions of pre-corroded D201-nZVI on Cr(VI) removal followed the order of Cl^−^ > SO_4_^2−^ > NO_3_^−^ > HCO_3_^−^ > H_2_O (control) > H_2_PO_4_^−^ > SiO_3_^2−^ in the same system (Figure 4d). To be simplified, the roles of six types of coexisting anions could be divided into two categories based on the positive or negative contribution to the reaction process.

**Figure 5:**
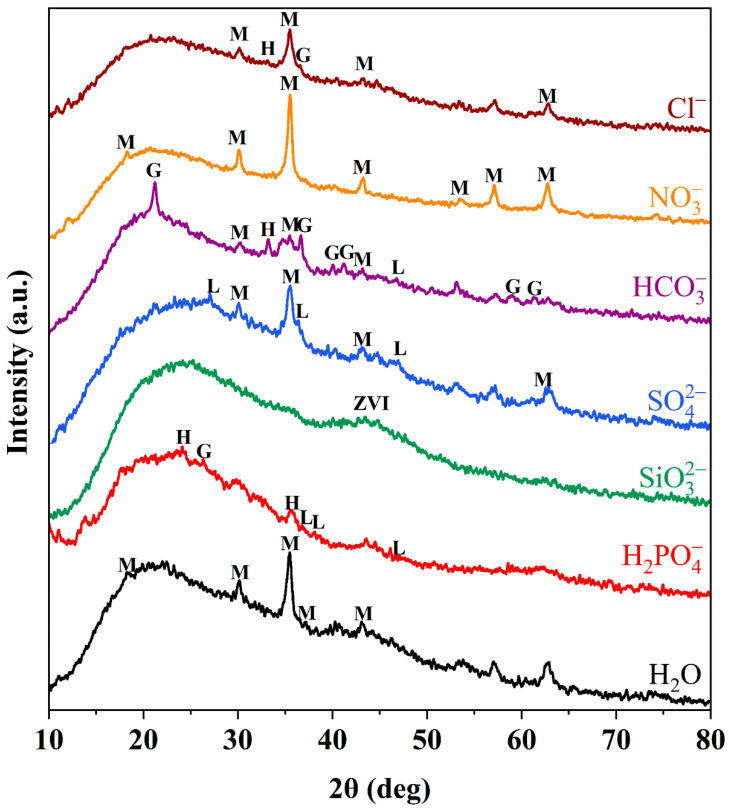
XRD patterns of D201-nZVI pre-corroded in the different system (M: magnetite (Fe_3_O_4_); H: hematite (Fe_2_O_3_); L: lepidocrocite (γ-FeOOH); G: goethite (α-FeOOH)).

Compared with aqueous solution, the effective peak intensity or number of corrosion products in the Cl^−^, SO_4_^2−^, NO_3_^−^, and HCO_3_^−^ system increased (Figure 5), indicating that the presence of Cl^−^, SO_4_^2−^, NO_3_^−^, and HCO_3_^−^ in the corrosion process promoted the generation of iron oxide to improve the Cr(VI) removal by pre-corroded D201-nZVI. The presence of iron oxide might be beneficial to increase the adsorption properties of pre-corroded D201-nZVI. In contrast, a lower corrosion degree was observed in the pre-corroded D201-nZVI from H_2_PO_4_^−^ and SiO_3_^2−^ solution, which was consistent with the results shown in Figure 4d. For instance, the effective peak intensity of iron oxide of D201-nZVI in the Cl^−^, NO_3_^−^, and SO_4_^2−^ system was higher than that of D201-nZVI in the HCO_3_^−^ system. The removal efficiency of D201-nZVI in the HCO_3_^−^ system was the lowest among the four types of anions. Generally, it was considered that the corrosion of ZVI occurred through the electrochemical process, with Fe^0^ dissolution as the anodic process and H_2_ evolution/O_2_ adsorption as the cathodic process. H_2_ evolution corrosion mainly occurred at pH < 4, while O_2_ adsorption mainly occurred when pH ranged from 4 to 10 [32]. Thus, it was reasonable that the iron corrosion was dominated by O_2_ adsorption corrosion in our study because the pHs of all the employed salt solutions were in the range of 4–10.

Cl^−^ could enter the center of ZVI and formed stable coordination complexes, which facilitated the formation of new reactive sites on the surface of pre-corroded D201-nZVI to promote the Cr(VI) removal [33]. Previous studies confirmed that SO_4_^2−^ could be incorporated into the crystal structure and the inner iron oxide/hydroxide double layer might promote the ZVI corrosion [32]. Green rust might be produced on the D201-nZVI surface during the corrosion process, which was conducive to the reduction of Cr(VI) removal by green Fe(II) rust [34]. Moreover, it has been reported that Fe_3_O_4_, the product of the NO_3_^−^ and Fe^0^, was the reason NO_3_^−^ promoted the Cr(VI) removal by pre-corroded D201-nZVI. Fe_3_O_4_ blocked the accumulation of corrosion products on the surface of pre-corroded D201-nZVI, and promoted the electron conduction, which contributed to the removal of Cr(VI) [35]. In addition, HCO_3_^−^ could have the process of ionization and hydrolysis in the solution simultaneously, preventing the rapid change in pH in the corrosion system. It could also produce a small quantity of H^+^, in turn slowing down the production of iron oxide/hydroxide on the pre-corroded D201-nZVI surface and promoting the Cr(VI) removal [31]. However, the generated Fe^2+^ in the presence of HCO_3_^−^ might react with HCO_3_^−^ to form iron carbonate FeCO_3_ on the pre-corroded D201-ZVI surface, which slowed down the electron transfer, reducing the Cr(VI) removal [36]. This might explain why the efficiency in the HCO_3_^−^ system was smaller than in the Cl^−^, SO_4_^2−^, and NO_3_^−^ system.

In contrast, the presence of H_2_PO_4_^−^ and SiO_3_^2−^ in the corrosion process inhibited the Cr(VI) removal by pre-corroded D201-nZVI. Phosphate reacted with Fe(II) to produce Fe_3_(PO_4_)_2_·8H_2_O precipitate coating on the D201-nZVI surface in the H_2_PO_4_^−^ system and its deposition might inhibit the further corrosion of ZVI. As a corrosion inhibitor, SiO_3_^2−^ could be attributed to its polymerization and coating effects in this study. During the corrosion process, silicate could be adsorbed on the corrosion products as a barrier and, gradually, silica polymer or amorphous solid phase was formed on the surface of D201-ZVI to inhibit corrosion [32], which might inhibit the removal efficiency of Cr(VI) by pre-corroded D201-nZVI.

Compared with aqueous solution, almost all cations in the corrosion process significantly contributed to the Cr(VI) removal by pre-corroded D201-nZVI. However, it was known that Cl^−^ strongly promoted the Cr(VI) removal, indicating the effect of Cl^−^ on the removal must be considered in the corrosion. Therefore, Na^+^ and K^+^ had insignificant effects on the Cr(VI) removal in the corrosion because of their solubilities. Compared with univalent cations, the presence of bivalent cations inhibited the Cr(VI) removal by pre-corroded D201-nZVI (Figure 4e). Previous studies indicated that Ca^2+^ might occupy the adsorption sites on the surface of D201-nZVI [37,38]. Similarly, a magnesium oxide–hydroxide passivation layer formed on the surface of D201-nZVI in the Mg^2+^ system, which reduced the number of reactive sites and had a negative effect on the Cr(VI) removal [37]. The Cr(VI) removal by the four types of pre-corroded D201-nZVI under the same ion conditions in the corrosion process. The removal efficiency followed the order of D1 > D2 > D3 > D4 (Figure 4d,e).

#### 3.3.5. Humic Acid

Natural organic matter, typically humus, is a constituent of water and soil [37]. Based on its solubility in acidic or alkaline solutions, humus, a brown amorphous polymer, can be divided into humic acid (HA, insoluble in acid), fulvic acid (FA, soluble in both acidic and alkaline solutions), and humin (insoluble in both acidic and alkaline solutions) [39]. The presence of HA in the corrosion slightly inhibited the Cr(VI) removal by pre-corroded D201-ZVI (Figure 4f). When other reductants were absent, HA had a weak reductivity. The occupation of the reactive sites by HA in the corrosion would inhibit the removal by pre-corroded D201-nZVI. Meanwhile, HA tend to form ZVI-HA complexes in the corrosion, which also inhibited the Cr(VI) removal [37,40]. The Cr(VI) removal by the four types of pre-corroded D201-ZVI still followed the order of D1 > D2 > D3 > D4 under the same HA concentration (Figure 4f).

### 3.4. Cr(VI) Removal Mechanism of Pre-Corroded D201-nZVI

**Figure 6:**
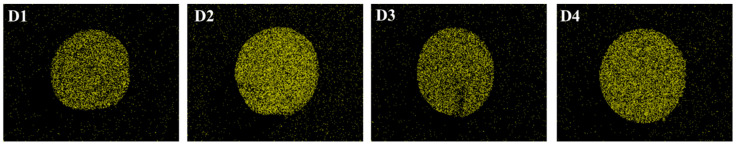
SEM-EDS spectra of elemental Cr mapping in the cross section of pre-corroded D1–D4 after reaction with Cr(VI).

The mechanism of Cr(VI) removal by pre-corroded D201-nZVI was explored based on SEM-EDS and XPS analyses. The SEM-EDS spectra of elemental Cr mapping in the cross section of pre-corroded D1–D4 after reaction with Cr(VI) indicated that the Cr element was well dispersed (Figure 6) and was different from the Fe element on the surface of pre-corroded D201-nZVI (Figure 1). It could be inferred that Cr(VI) removal by pre-corroded D201-nZVI included the reduction of nZVI and adsorption of resin through the ion exchange. ZVI occupied the reactive sites of the internal resin with the increase in the uniformity of the iron distribution on the resin, which might weaken the adsorption of resin. Meanwhile, the improvement in ZVI uniformity on the resin promoted contact with ZVI and Cr(VI), which might strengthen the reduction of ZVI.

**Figure 7:**
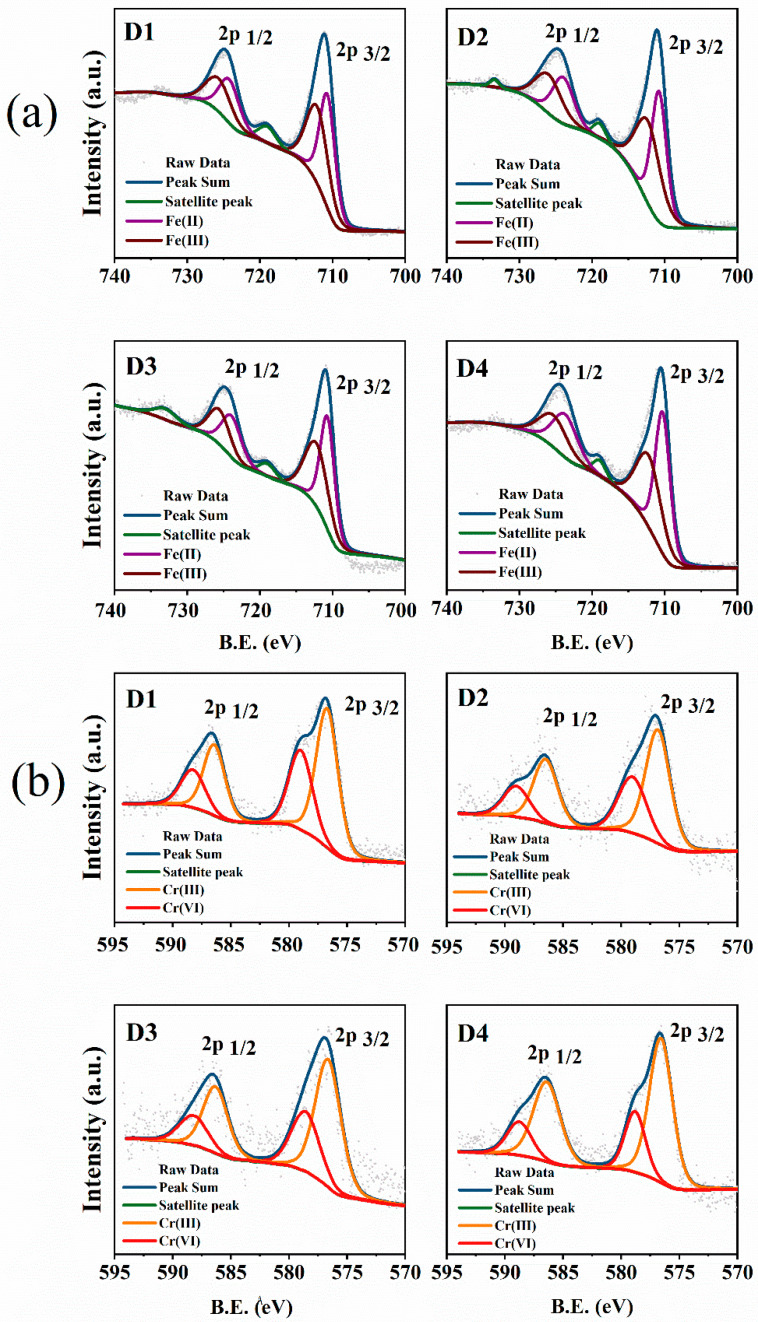
Fe2p (**a**) and Cr2p (**b**) high-resolution XPS spectra of pre-corroded D1–D4 after reaction with Cr(VI).

XPS analysis was performed to further investigate the chemical compositions and the oxidation states of Cr and Fe on the surface of pre-corroded D1–D4 after reaction with Cr(VI). There were elements Fe, C, O, and N that could be detected before the reaction with Cr(VI) on the survey scan of the XPS spectra (Figure S2a). However, the new peak appeared at 577 and 586 eV, corresponding to the element Cr, after reaction with Cr(VI), which indicated that Cr was successfully immobilized on the surface of the pre-corroded D201-nZVI (Figure S2b). Moreover, the high-resolution XPS spectra of Fe 2p and Cr 2p regions are shown in Figure 7. A survey spectrum of the Fe 2p levels after reaction with Cr(VI) (Figure 7a) showed photoelectron peaks at 724.20 and 725.50 eV corresponding to the binding energy of Fe 2p1/2, while the binding energies at 710.30 and 712.30 eV in the high-resolution Fe 2p3/2 spectra represented Fe^2+^ and Fe^3+^ in FeCr_2_O_4_, and Fe_3_O_4_, respectively [41], which also implied that ZVI was present on the surface of D201 and was covered by a layer of iron oxides.

**Figure 8:**
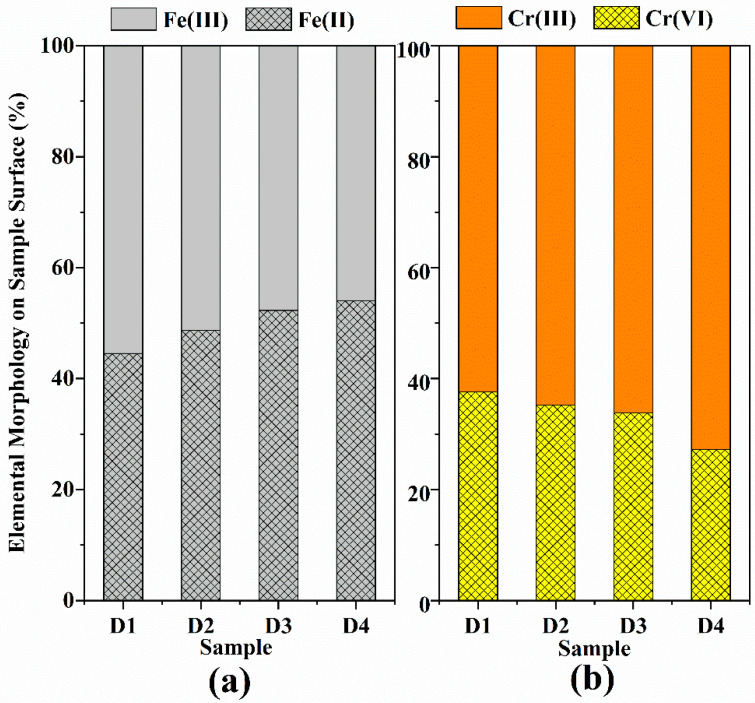
Oxidation states of Fe (**a**) and Cr (**b**) immobilized onto pre-corroded D1–D4 after reaction with Cr(VI).

Furthermore, the reduction of Cr(VI) was apparent from the Cr 2p XPS spectra for D201-nZVI after reaction with Cr(VI) (Figure 7b). The Cr 2p3/2 peaks at 576.80 and 579.00 eV correspond to Cr(OH)_3_ and K_2_CrO_4_, respectively [42], which suggested that both adsorption and redox reaction contributed to the removal of Cr(VI) and coprecipitates were formed during Cr(VI) adsorption and reduction. After reaction with Cr(VI), 37.63%, 35.23%, 33.84%, and 27.20% of D201-nZVI surface from D1 to D4 remained as Cr(VI), while 62.37%, 64.77%, 66.16%, and 72.80% was converted to Cr(III) (Figure 8b and Table S2). Most of the chromium adsorbed on the surface of D201 was reduced to Cr(III), with only less remaining as the Cr(VI) form. These results also revealed that both adsorption and redox reaction contributed to the removal of Cr(VI) from aqueous solution. Meanwhile, the peak areas corresponding to the Fe(II) from D1 to D4 were 44.53%, 48.73%, 52.33%, and 54.06%, respectively (Figure 8a and Table S1), indicating that the ZVI acted as an electron donor and contributed to the reduction of Cr(VI) to Cr(III) on the surface of D201.

XPS results confirmed the combined effects of adsorption and reduction achieved by modification of D201 with ZVI to improve Cr(VI) removal ability of D201-nZVI, and the corresponding mechanism might involve the generation of nascent Fe(II) and electron transfer from the corrosion evolution of nZVI. The proportion of Cr(III) gradually increased with the increase in the uniformity of the iron distribution on the resin, indicating that the increase in iron distribution uniformity was favorable for reduction of pre-corroded D201-nZVI. Moreover, the ratio of Cr(III) on the pre-corroded D201-nZVI surface was much higher than that of Cr(VI), which confirmed that the reduction was the main removal mechanism of pre-corroded D201-nZVI. The surface-bound Fe(II) on the surface of pre-corroded D201-nZVI and the corrosion products might increase the surface positive charge in favor of adsorption and reduction of Cr(VI). Generally, the Cr(VI) removal mechanism by pre-corroded D201-nZVI included the direct/indirect reduction of nZVI, adsorption of iron oxide/hydroxide, and adsorption of D201 resin.

## 4. Conclusions

In this study, the effect of spatial distribution of nZVI on the corrosion behavior of D201-nZVI and the Cr(VI) removal by the corresponding pre-corroded D201-nZVI were systematically investigated. The Cr(VI) removal efficiency by the four types of pre-corroded D201-nZVI followed the order of D1 > D2 > D3 > D4 at the same corrosion condition. Acidic or alkaline conditions and all coexisting ions except SiO_3_^2−^ and H_2_PO_4_^−^ enhanced the corrosion of nZVI to different extents during the corrosion process. The effect of coexisting anions of pre-corroded D201-nZVI on Cr(VI) removal followed the order of Cl^−^ > SO_4_^2−^ > NO_3_^−^ > HCO_3_^−^ > H_2_O > H_2_PO_4_^−^ > SiO_3_^2−^ in the same system. Meanwhile, humic acid in the corrosion system slightly decelerated Cr(VI) removal. The corrosion products derived from nZVI, including magnetite, hematite, lepidocrocite, and goethite, were identified by XRD. The dominant corrosion products were found to be highly dependent on the type of coexisting ions, and the roles of different coexisting anions on nZVI corrosion were also elucidated. The distributions of Fe and Cr oxidation states immobilized onto pre-corroded D201-nZVI were revealed by XPS analysis, and we found that the nZVI acted as an electron donor and contributed to the reduction of Cr(VI) to Cr(III) on the surface of D201, and the increase in iron distribution uniformity was favorable for the reduction of pre-corroded D201-nZVI. The mechanisms of Cr(VI) removal by pre-corroded D201-nZVI were proved to be a comprehensive process involving the direct/indirect reduction of nZVI, adsorption of iron oxide/hydroxide, and adsorption of D201 resin, and reduction was the main removal mechanism of pre-corroded D201-nZVI. This study provides insight into the selection of nZVI composites with different spatial distribution of pre-corroded nZVI under different coexisting solutions for practical water remediation and promotes the understanding of the fate of nZVI−Cr(VI) reaction products in the natural environment.

## Figures and Tables

**Table 1:** Kinetic parameters of the pseudo-first-order and pseudo-second-order model for Cr(VI) removal by fresh and pre-corroded D201-nZVI.

			Pseudo-First-Order Kinetics			Pseudo-Second-Order Kinetics		
Corrosion Time (h)	Samples	Q_e_, exp (mg/g)	k_1_ (min^−1^)	Q_e_ (mg/g)	R^2^	k_2_ (g mg^−1^ min^−1^)	Q_e_ (mg/g)	R^2^
0	D1	130.58	8.95 × 10^−3^	132.72	0.9978	5.85 × 10^−3^	170.91	0.9988
	D2	109.52	1.20 × 10^−2^	109.23	0.9817	7.59 × 10^−3^	131.75	0.9880
	D3	96.18	3.76 × 10^−2^	87.35	0.9610	1.02 × 10^−2^	97.62	0.9723
	D4	101.10	2.73 × 10^−2^	94.67	0.9745	9.43 × 10^−3^	106.06	0.9899
48	D1	134.61	8.01 × 10^−3^	138.77	0.9911	5.52 × 10^−3^	181.04	0.9929
	D2	116.51	1.18 × 10^−2^	114.33	0.9908	7.18 × 10^−3^	139.29	0.9954
	D3	97.70	1.75 × 10^−2^	92.42	0.9710	9.42 × 10^−3^	106.16	0.9854
	D4	104.21	1.95 × 10^−2^	96.45	0.9461	9.19 × 10^−3^	108.82	0.9753

## Data Availability

Data can be available upon request from the authors.

## Supplementary Materials

The supplementary materials can be download at the following link: https://www.mdpi.com/article/10.3390/nano12030494/s1. Table S1. The properties of four types of fresh D201-nZVI. Table S2. Oxidation states proportion of Fe and Cr immobilized onto pre-corroded D201-nZVI surface after reaction with Cr(VI). Figure S1. Effect of the Cl^−^ concentration on Cr(VI) removal by pre-corroded D201-nZVI. Figure S2. XPS spectra of pre-corroded D201-nZVI (a) before (b) after reaction with Cr(VI).
