# Supplementary material for: Effect of Spatial Distribution of nZVI on the Corrosion of nZVI Composites and Its Subsequent Cr(VI) Removal from Water
Source: Nanomaterials (Basel). 2022 Jan 30;12(3):494. doi: 10.3390/nano12030494 (PMC8840039; doi:10.3390/nano12030494)
Supplement: Supplementary file 1 [file nanomaterials-12-00494-s001.zip › nanomaterials-1494669-SI.pdf]

Supplementary Materials

Effect of Spatial Distribution of nZVI on the Corrosion of nZVI Composites and Its Subsequent Cr(VI) Removal from Water

Yixuan Li, Shuangqiu Huang, Yaqin Song, Xinfang Zhang, Sijia Liu and Qiong Du *

School of Engineering, China Pharmaceutical University, Nanjing, 211198, China; liyixuan_1117@163.com (Y.L.); 2111904034@e.gzhu.edu.cn (S.H.); songyq_11@126.com (Y.S.); zhxf20@163.com (X.Z.); liusijia_98@163.com (S.L.)

* Correspondence: duqiong116@163.com; +86-25-8618-5190

Table S1. The properties of four types of fresh D201-nZVI.

Materials	D1	D2	D3	D4
color	black	black	black	black
dimeter (mm)	0.7-0.8	0.7-0.8	0.7-0.8	0.7-0.8
surface area (m^2 g^{-1})	9.75	14.70	15.63	16.40
Fe content (%)	13.81	13.36	14.05	13.75

Table S2. Oxidation states proportion of Fe and Cr immobilized onto pre-corroded D201-nZVI surface after reaction with Cr(VI).

Samples	Proportion of oxidation states on D201-nZVI Surface(%)			
	Fe(II)	Fe(III)	Cr(VI)	Cr(III)
D1	44.53	55.47	37.63	62.37
D2	48.73	51.27	35.23	64.77
D3	52.33	47.67	33.84	66.16
D4	54.06	45.94	27.20	72.80

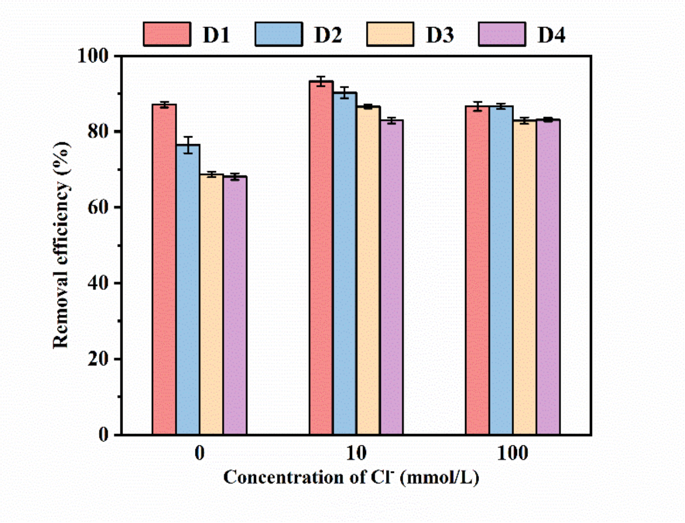

Figure S1. Effect of the Cl^- concentration on Cr(VI) removal by pre-corroded D201-nZVI.

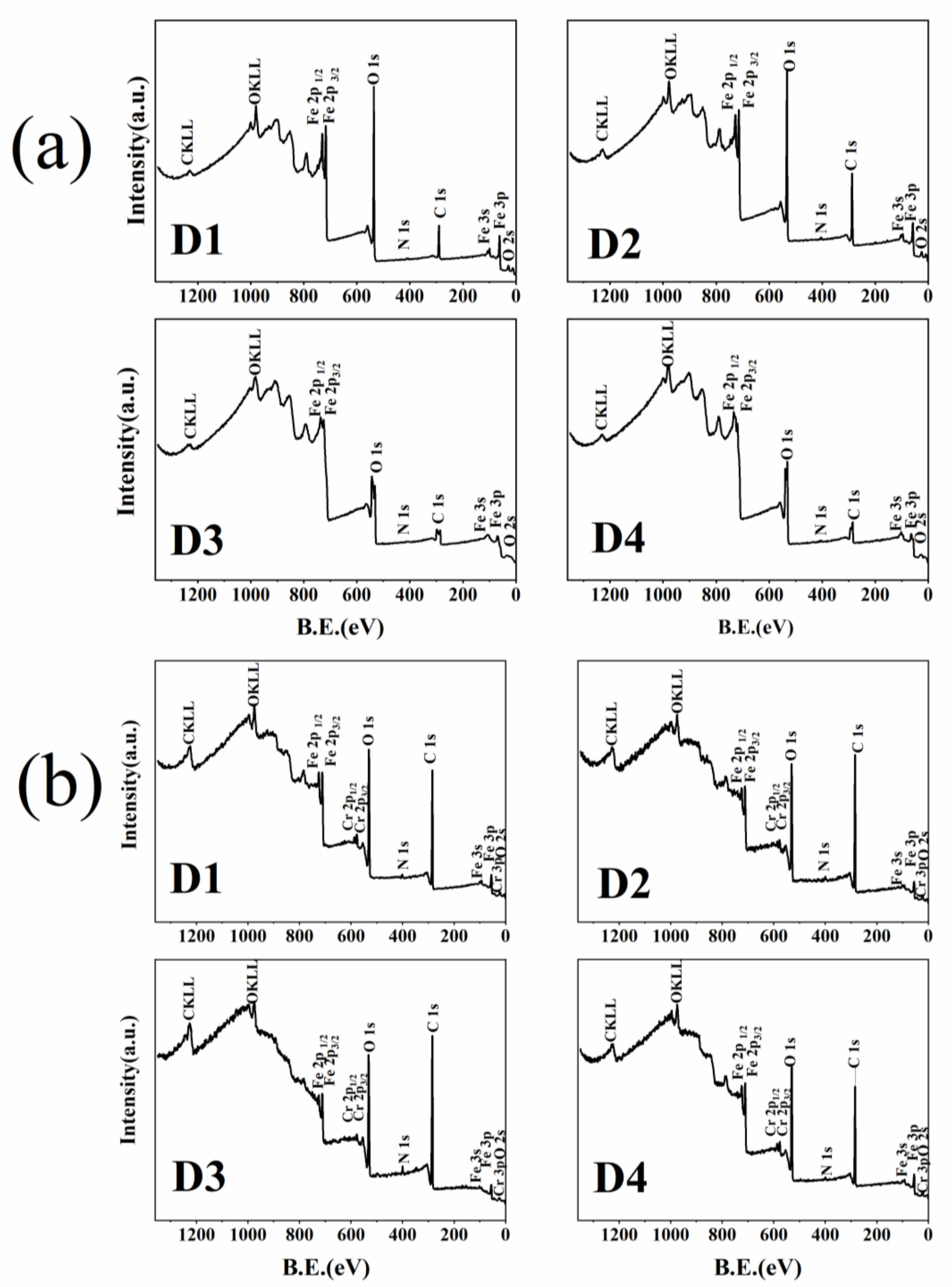

Figure S2. XPS spectra of pre-corroded D201-nZVI (**a**) before (**b**) after reaction with Cr(VI).
